# Preparation and application of conducting polymer/Ag/clay composite nanoparticles formed by *in situ* UV-induced dispersion polymerization

**DOI:** 10.1038/srep20470

**Published:** 2016-02-03

**Authors:** Limin Zang, Jianhui Qiu, Chao Yang, Eiichi Sakai

**Affiliations:** 1 Department of Machine Intelligence and Systems Engineering, Faculty of System Science and Technology, Akita Prefectural University, Yurihonjo 015-0055, Japan; 2 State Key Laboratory Breeding Base of Nonferrous Metals and Specific Materials Processing, College of Material Science and Engineering, Guilin University of Technology, Guilin 541004, China

## Abstract

In this work, composite nanoparticles containing polypyrrole, silver and attapulgite (PPy/Ag/ATP) were prepared via UV-induced dispersion polymerization of pyrrole using ATP clay as a templet and silver nitrate as photoinitiator. The effects of ATP concentration on morphology, structure and electrical conductivity were studied. The obtained composite nanoparticles with an interesting beads-on-a-string morphology can be obtained in a short time (10 min), which indicates the preparation method is facile and feasible. To explore the potential applications of the prepared PPy/Ag/ATP composite nanoparticles, they were served as multifunctional filler and blended with poly(butylene succinate) (PBS) matrix to prepare biodegradable composite material. The distribution of fillers in polymer matrix and the interfacial interaction between fillers and PBS were confirmed by scanning electron microscope, elemental mapping and dynamic mechanical analysis. The well dispersed fillers in PBS matrix impart outstanding antibacterial property to the biodegradable composite material as well as enhanced storage modulus due to Ag nanoparticles and ATP clay. The biodegradable composite material also possesses modest surface resistivity (10^6^ ~ 10^9^ Ω/◻).

Recently different matrices decorated by Ag nanoparticles like clay, metal oxides, carbon materials and polymers have been developed in the fields of food packaging, biomedical applications and photocatalysis, since they possess unusual antibacterial activity and photoelectrical properties^1^,^2^,^3^,^4^. Owing to wide range of conductivities over many orders of magnitude, redox property, light-weight and ease of handling, conducting polymers have attracted considerable attention and exhibited promising applications in many fields like energy storage, sensors, electromagnetic shielding, corrosion, microelectronics, electrochromics, etc^5^,^6^ The consequent related development of incorporating Ag nanoparticles into conducting polymers is of great interest in virtue of their synergetic physicochemical properties based on the conducting polymer and Ag nanoparticles^7^. Particularly, PPy/Ag composites have received significant attention because of their excellent biocompatibility, antibacterial activity, electro-catalytic activity and electrochemical property. Generally speaking, several strategies are available for preparing of PPy/Ag composites: (i) Dispersion of the Ag nanoparticles on the surface of a polymer or in a polymer matrix;^8^ (ii) *In situ* polymerization of PPy around the Ag nanoparticles;^9^,^10^ (iii) Reduction of silver ions using the polymer or monomer as a reducing agent^11^,^12^,^13^. Utilization of photochemical reactions (photoexcitation of the monomer itself or photocatalytic systems) to synthesize conducting polymers is of note^14^,^15^,^16^. It has been demonstrated of late that photopolymerization of PPy in the presence of silver nitrate (AgNO_3_) is a convenient and effective method to incorporate Ag particles into PPy^5^,^17^. Singh and co-workers prepared free standing PPy/Ag nanocomposite films which were used as electrode material for supercapacitors by photopolymerization of pyrrole using AgNO_3_ as photosensitizer in water for 150 min. Two films were formed at the bottom of the beaker and at the air–water interface^18^. By selection of suitable substrate, it might be a remarkable and useful approach to fabricate satisfactory materials for energy storage, catalysis and sensors. Singh *et al*. prepared PPy/Ag nanocomposite films on N-(3-trimethoxysilylpropyl)pyrrole modified biaxially oriented polyethylene terephthalate by photopolymerization for 15 ~ 180 min. The films prepared at certain conditions (Pyrrole : AgNO_3_ = 0.5 M : 0.1 M, UV exposure time: 120 min) showed gas sensing properties^17^. Gerbaldi *et al*. reported a method to prepare PPy/Ag/methacrylate polymer film by UV-induced polymerization using AgNO_3_ as metal precursor and oxidant for pyrrole and Darocur 1173 as photoinitiator for bisphenol A ethoxylate dimethacrylate. During polymerization, Ag^+^ and pyrrole migrated toward both surfaces to form a metallo-polymer capacitor^19^.

In terms of good electrical conductivity, good environmental stability and ease of preparation, PPy is deemed one of the most important conducting polymers^20^. However, like other typical conducting polymers, PPy suffers low mechanical strength, brittleness and poor processability, leading to finite applications. A workable method to solve these limitations is compounding with inorganic component. By *in situ* polymerization of pyrrole or coating PPy on the surface of inorganic substrate, novel composites with unique properties could be achieved. Mechanical properties of the substrates as well as electrical conducting properties of PPy are inherited, and at the same time unprecedented properties differing from their individual component based on synergy effect may also reveal^21^,^22^. Up to now, numerous inorganic/PPy composites that contain various inorganic matrices have been prepared, including mesoporous silica, TiO_2_, reduced graphene oxide, clay, etc^23^,^24^,^25^,^26^. Significantly, polymerization of pyrrole in the presence of inorganic colloidal particles is of particular interest because it can form well-defined and stable core-shell composites. Zhao *et al*. used octahedral PbS nanocrystals colloid to synthesize PbS/PPy core-shell nanocomposites via *in situ* chemical oxidation polymerization of pyrrole^27^. Metallic Cu nanoparticles with a size of 27.6 ± 11.1 nm coated with PPy were obtained by polymerizing pyrrole in an aqueous colloid solution of the Cu nanoparticles^28^. Silica colloid were also used to prepare silica/PPy nanocomposites in a soap-free system^29^,^30^. Natural clay mineral attapulgite (ATP) which has unique fibrous morphology and large surface area, can disperse easily in aqueous to form a stable colloidal network under modest conditions. These characteristics of ATP attract our attention considering its potential to establish stable well-defined fibrous composite by coating PPy on the surface of ATP.

In view of the above possibilities, *in situ* UV-induced dispersion polymerization technology was employed to generate Ag particles and ATP as the functional components to prepare PPy composites in this work. In this approach, ATP clay dispersed in aqueous played the role as a templet, and AgNO_3_ was used as photoinitiator. Immediately after exposure to UV light, silver ions were excited and then oxidized pyrrole to form PPy while they were reduced to metallic Ag. In other words, PPy and Ag nanoparticles were produced synchronously to form one-dimensional PPy/Ag/ATP composites when exposed to UV light. The UV-induced polymerization is rapid and all the reactions can be completed within 10 min. Furthermore, the incorporated ATP could not only enhance the mechanical strength but also work as a templet to obtain fibrous composites, and meanwhile the existence of Ag particles could also broaden the range of applications in catalysts, analysis, antimicrobials, etc^31^,^32^,^33^. One application example of the obtained PPy/Ag/ATP composite nanoparticles was explored in this work. They were served as multifunctional filler to prepare biodegradable polymer-based composite material. Poly(butylene succinate) (PBS) that possesses excellent biodegradability, good processability, high thermal and chemical resistance^34^ was chosen as the matrix, and the incorporated composite nanoparticles could play multiple parts as reinforced filler, antistatic and antibacterial agent.

## Results

### Preparation of PPy/Ag/ATP composite nanoparticles

**Figure 1:**
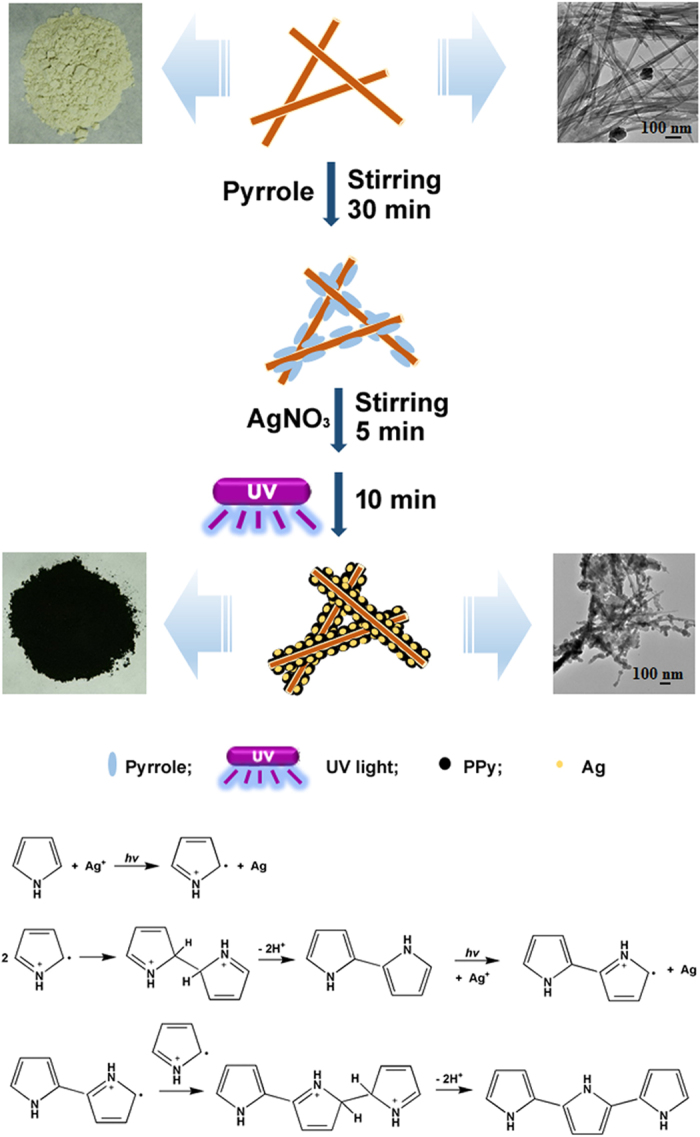
The fabrication process of PPy/Ag/ATP composite nanoparticles by UV-induced polymerization.

UV-induced dispersion polymerization technique was employed to prepare PPy/Ag/ATP composite material with an interesting beads-on-a-string morphology, where UV light was used as energy source, fibrous ATP clay served as a templet, and AgNO_3_ acted as photoinitiator as well as a source of Ag ions. ATP used in this work can be easily dispersed in water and form a colloidal network with modest shear. Pyrrole partially adsorbed onto the ATP particles along with vigorous ultrasonic prior to polymerization after it was added to ATP colloid^29^. When AgNO_3_ solution was introduced, tiny amounts of Ag^+^ were thought to be located on the surface of ATP clay owing to the electrostatic interactions between Ag^+^ and ATP clay. Upon exposure to UV light, the polymerization of pyrrole and the reduction of Ag^+^ were conducted simultaneously. The fabrication process by UV-induced dispersion polymerization is illustrated in Fig. 1. The pristine ATP clay with a light cream colour became black after UV-induced polymerization of pyrrole. When the suspension was exposed to the UV light, Ag^+^ that served as an oxidant received electrons from the excited pyrrole monomer, resulting in the formation of pyrrole radical cations (oxidation) and the reduction of Ag^+^ to metal. Then two pyrrole radical cations underwent coupling to form a dimer (dimerization) and the dimers underwent the same oxidation as pyrrole monomer described above to form a dimer radical cations. These radical cations can further react with the pyrrole radical cations to form the PPy chain, known as chain growth^35^,^36^.

### Morphology

**Figure 2:**
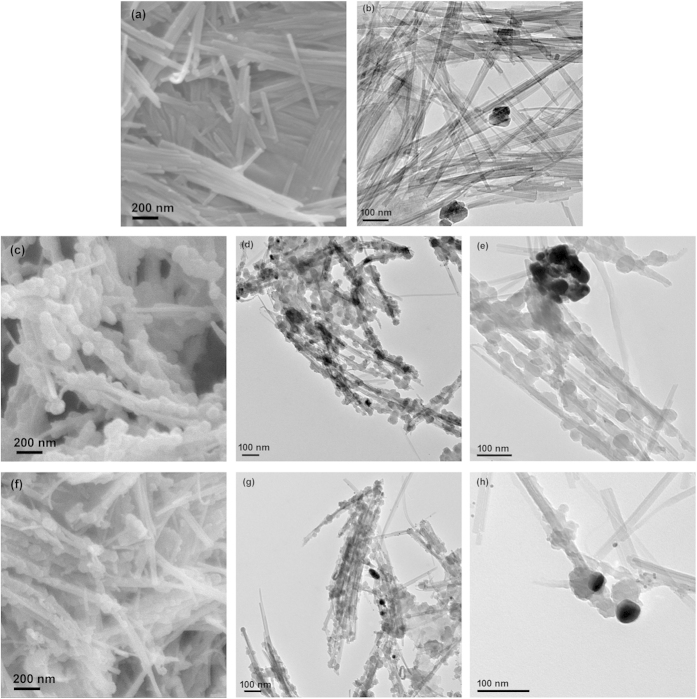
SEM (**a**) and TEM (**b**) images of raw ATP clay; SEM (**c**) and TEM (**d**,**e**) images of PPy/Ag/clay composite nanoparticles (mass ratio of ATP to pyrrole was 20:100); SEM (**f**) and TEM (**g**,**h**) images of PPy/Ag/ATP composite nanoparticles (mass ratio of ATP to pyrrole was 50:100).

The morphology of ATP and PPy/Ag/ATP composite nanoparticles were observed by SEM and TEM, and the images are shown in Fig. 2. The SEM and TEM image indicated that the ATP clay presented fibrous morphology with an average diameter of 20 nm and length of 0.5 ~ 1 μm, and the surface of the ATP clay was smooth. For PPy/Ag/ATP composite nanoparticles, a thin layer PPy and a great deal of granular PPy nanoparticles were coated on the surface of ATP, forming a beads-on-a-string structure. In addition, a few number of metallic Ag with a size of a few nanometers also presented on the surface of ATP, which might stem from the reduction of Ag^+^ that adsorbed on the surface of ATP through electrostatic interaction. The SEM image with lower magnification revealed that, besides Ag nanoparticles, Ag aggregates of submicron/micron-size also appeared. Moreover, the aggregation of ATP became obvious and many ATP clay didn’t coat with PPy when the concentration of ATP was high.

### Structure analyses

**Figure 3:**
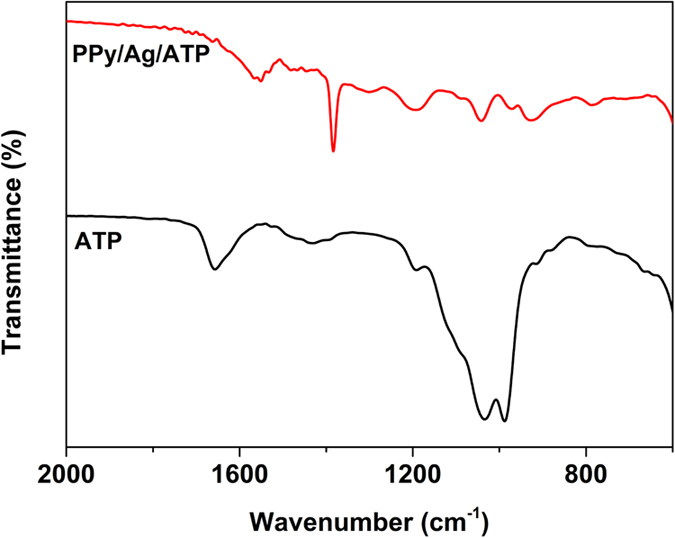
FTIR spectra of ATP and PPy/Ag/ATP nanocomposite (S-3).

The ATP and as prepared PPy/Ag/ATP composite nanoparticles were characterized by FTIR spectroscopy (Fig. 3). Characteristic peaks corresponding to both PPy and ATP clay appeared in the spectrum. The peaks at 1550 and 1465 cm^−1^ was assigned to C=C and C-N stretching vibration in the pyrrole ring, respectively. The peaks presented at 1299 and 786 cm^−1^ was due to =C-H in-plane deformation and C-H wagging vibration, respectively^37^,^38^. The peak at 1193 cm^−1^ was associated with the characteristic of ATP that didn’t appear in any other clay silicate. Moreover, the characteristic peak of NO_3_^−^ at 1385 cm^−1^ also arose in the spectrum for PPy/Ag/ATP composite nanoparticles, indicating PPy has been doped by NO_3_^−^39.

**Figure 4:**
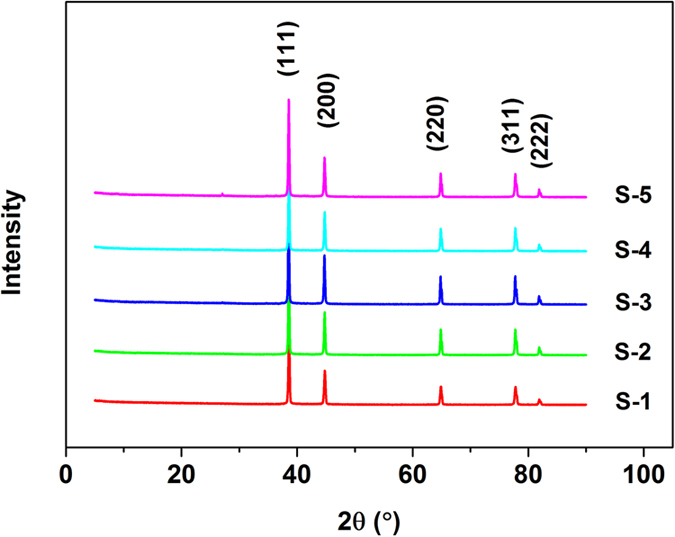
XRD patterns of ATP and PPy/Ag/ATP nanocomposites.

The XRD patterns of PPy/Ag/ATP composite nanoparticles with varied content of ATP are presented in Fig. 4. Five diffraction peaks appeared at 38.5°, 44.7°, 64.8°, 77.7° and 81.7°, corresponding to (111), (200), (220), (311) and (222) planes of cubic phase of Ag respectively^40^. The maximum peak intensity of (111) diffraction for Ag was used to calculate the average crystalline size using Scherrer’s equation and the results were listed in Table 1. The average crystalline size of Ag was about 40 nm, indicating that Ag^+^ ions have been reduced to metallic Ag particles with nano-scale upon UV-induced polymerization. Additionally, the much higher intensity of peaks for metallic Ag nanoparticles cover the peaks for ATP and PPy.

### XPS analysis

**Figure 5:**
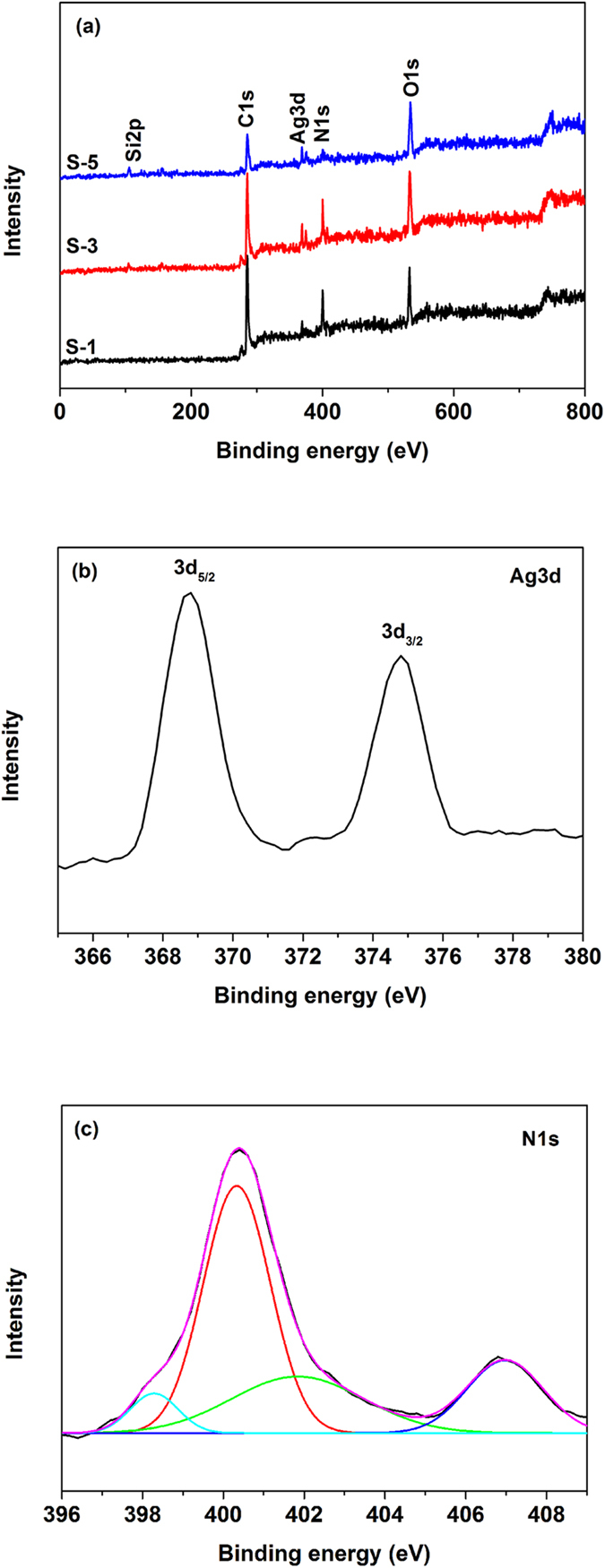
XPS survey spectra for PPy/Ag/ATP nanocomposites (**a**) and high resolution spectra of S-3 for Ag3d (**b**) and N1s (**c**).

XPS measurements were performed to investigate the chemical composition and the chemical state of Ag and N in the PPy/Ag/ATP composite nanoparticles. Figure 5(a) shows the XPS survey spectra for PPy/Ag/ATP composite nanoparticles (S-1, S-3 and S-5). All the samples showed peaks for Si2p (105 eV), C1s (285 eV), Ag3d (368-374 eV), N1s (400 eV), and O1s (532 eV)^41^. The Ag3d and N1s high resolution spectrum of S-3 is shown in Fig. 5(b,c), respectively. Ag3d doublet centered at 368 and 374 eV were attributed to Ag3d_5/2_ and Ag3d_3/2_, confirming that metallic Ag particles have been generated during the UV-induced polymerization process. Concerning N1s high resolution spectrum, 4 kinds of chemical state of N in the composite can be confirmed. The main peak at 400.3 eV was associated with the neutral N in PPy and the peak at 401.8 eV was attributed to N^+^ in PPy ring that represented doped PPy. Counterions NO_3_^−^ also presented at 406.9 eV, which played the role as dopants. The shoulder peak at 398.3 eV was attributed to C=N component^18^. The doping level of PPy in the composite was determined by N^+^/(neutral N and N^+^) atomic ratio and value was about 0.29. It is interesting to note that the N/Ag atomic ratio gradually decreased with increasing feeding concentration of ATP, although the initial feeding ratio of pyrrole/AgNO_3_ was kept as a constant (Table 2).

### Electrical conductivity

The electrical conductivity of the nanocomposite with varied concentration of ATP clay is summarized in Table 1. The maximum electrical conductivity was 0.442 S/cm for S-3, which was comparable with other reported values for similar PPy/Ag/insulated substrate composites^5^,^7^,^13^,^19^. The electrical conductivity of the nanocomposite that was attributed to the presence of doped PPy and metallic Ag particles, increased firstly and then decreased with the increment of ATP concentration. For S-5 (feeding mass ratio of ATP to pyrrole was 50:100), the abrupt increment in electrical conductivity appeared.

### Potential application instance

UV-induced polymerization of pyrrole in the presence of AgNO_3_ and ATP clay to prepare conducting Ag composite is absorbing because: (1) Unlike other time consuming processes, UV-induced polymerization can be conducted in relative short time. In our work, it only took 10 min to complete the polymerization of pyrrole. (2) The resulting nanocomposites composing of PPy, Ag and ATP show unique electrical, antibacterial and mechanical properties based on synergy effect and exhibit a wide range of potential applications. As an application instance, the as prepared PPy/Ag/ATP composite nanoparticles were compounded with biodegradable polymer (PBS), and the obtained PBS-based composite material was expected to show some interesting properties and more extensive application prospect.

**Figure 6:**
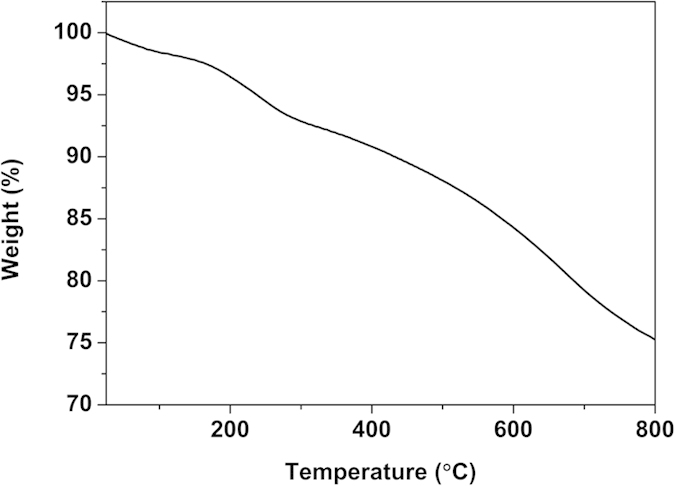
TGA of PPy/Ag/ATP nanocomposite (S-3).

Considering the PBS-based composite material should be processed at high temperature, the thermal stability of the PPy/Ag/ATP composite nanoparticles (S-3) was investigated by TGA and the curve is shown in Fig. 6. The initial weight loss of ~2% below 120 °C was mainly due to the expulsion of adsorbed water. The following weight loss from ~185 °C was caused by the degradation of PPy chains and dehydration of ATP (coordination water and dehydroxylation)^42^. Based on the above results, commercial PBS and PPy/Ag/clay composite nanoparticles were dried at 80 °C for 12 h before melt blending to avoid the influence of adsorbed water on the properties of PBS-based composite material. The thermal stability of PPy/Ag/ATP composite nanoparticles could ensure that they can withstand the melt blending temperature of 135 °C.

**Figure 7:**
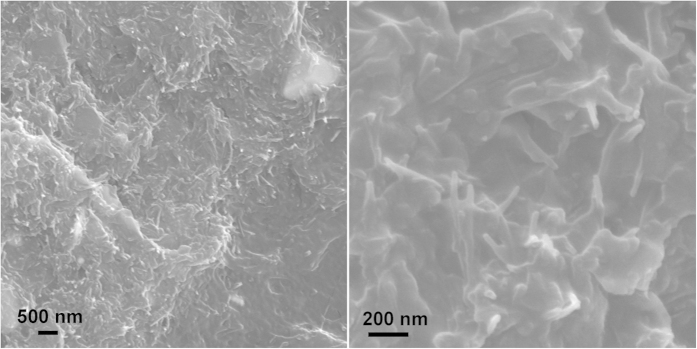
SEM images of fracture surface of 15% PBS composite at different magnifications.

**Figure 8:**
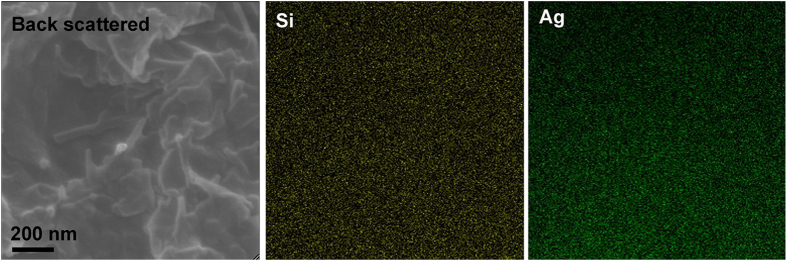
Back scattered electron image and elemental mapping of Si and Ag in the 15% PBS composite.

The fracture surface of composite specimen was characterized by SEM. From the SEM image with higher magnification it’s observed that ATP dispersed uniformly in the matrix and wasn’t pulled out (Fig. 7). However, as said earlier, upon UV-induced polymerization, not only nano-sized but also a small number of submicron/micron-sized Ag particles are formed, resulting in the presence of Ag aggregates of micron size in the PBS matrix. In order to further analyze the distribution of ATP clay and Ag, elemental mapping of Si and Ag is recorded in Fig. 8. The homogeneous brightness and distribution proved the even distribution of Ag and ATP clay, which can offer antibacterial property and enhance mechanical property of the composite material.

**Figure 9:**
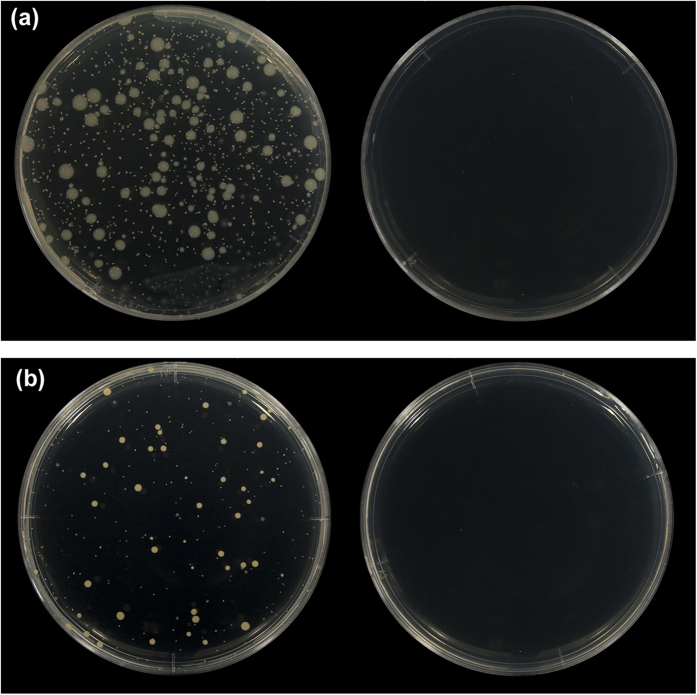
Antibacterial activity of 15% PBS composite against *Escherichia coli* (**a**) and *Staphylococcus aureus* (**b**) (left: pure PBS; right: PBS composite).

The even distribution of Ag nanoparticles in PBS matrix is conspicuous for the potential antibacterial property^43^,^44^. The antibacterial activity of pure PBS and PBS-based composite material against two typical bacterials *Escherichia coli* and *Staphylococcus aureus* were carried out under same conditions following the standard JIS Z 2801: 2010^45^. From the results as shown in Fig. 9, we can visually identify that the PPy/Ag/ATP nanoparticles filled PBS composite material exhibited excellent antibacterial activity against both *Escherichia coli* and *Staphylococcus aureus*, whereas, the antibacterial property of pure PBS was rather poor. The antibacterial activity R for PBS-based composite material against *Escherichia coli* and *Staphylococcus aureus* was 5.8 and 5.4, respectively, which was much higher than the standards value of antibacterial activity for plastics (R > 2).

**Figure 10:**
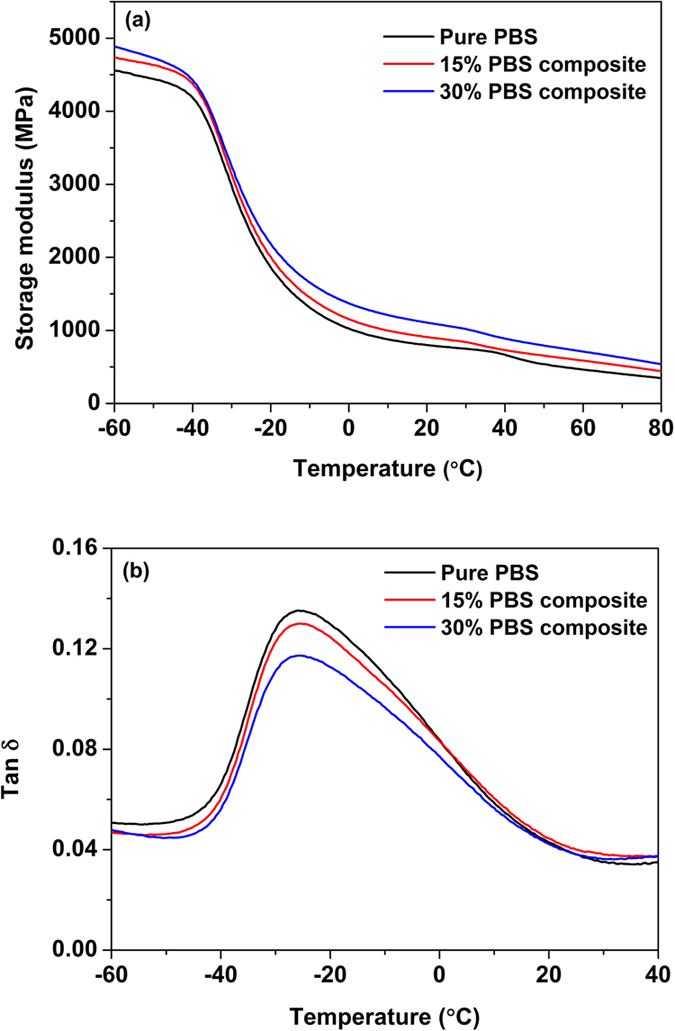
Temperature dependence of storage modulus (**a**) and tan δ (**b**) for pure PBS and PBS-based composite materials.

To estimate the interactions between the PPy/Ag/ATP composite nanoparticles and PBS matrix, DMA measurements were performed. Figure 10 displays the temperature dependence of storage modulus and tan δ of pure PBS and PBS-based composites. The storage modulus of the PBS-based composite increased with the increasing filler content. For example, the storage modulus of 30% PBS composite increased to 4891 MPa at the glassy region (−60 °C), whereas the value for pure PBS was 4560 MPa. However, the shift of tan δ peak of PBS composite was negligible, meanwhile, the height of the peak decreased with the addition of PPy/Ag/ATP composite nanoparticles.

The surface resistivity of 15% PBS composite was ~10^9^ Ω/◻, and further reached to ~10^6^ Ω/◻ when the filler content was 30%. The good surface resistivity of the composite specimen stems from the PPy layer and metallic Ag nanoparticles that coated on the surface of ATP clay.

## Discussion

PPy/Ag/ATP composite nanoparticles were successfully prepared by UV-induced dispersion polymerization with the help of AgNO_3_. Our work is compared with other reports that used AgNO_3_ as an oxidant to perform polymerization of pyrrole with or without UV light^13^,^15^,^17^,^18^,^19^. Significantly, in this work UV-induced polymerization of pyrrole using AgNO_3_ as photoinitiator in ATP colloid can be finished in 10 min with high yield (~80%), demonstrating this strategy is facile and efficient. The high power of UV light (1000 W) may be responsible for the fast reaction rate. In the absence of UV light, the polymerization is time-consuming, although Ag^+^ has the standard reduction potential (0.799 V) similar to Fe^3+^ (0.769 V). Some researchers applied AgNO_3_ as an oxidant to conduct polymerization of pyrrole, but they generally took ~24 h without the help of UV light^13^,^15^. It may be that oxidative polymerization of pyrrole is kinetically very slow for Ag^+^^11^.

The beads-on-a-string morphology observed by SEM and TEM (Fig. 2) can be explained by the formation mechanism as being supposed in Fig. 1. Colloidal network of ATP is conducive to stabilize the mixture system and fibrous ATP clay serves as a templet. The relative high oil absorption value (100 mL/100g) and Si-OH groups present on the ATP are responsible for adsorbing partial pyrrole monomer. During the UV-induced polymerization process, the pyrrole monomer adsorbed on the surface of ATP formed pyrrole radical cations and dimers radical cations (and/or higher oligomers radical cations), and then underwent chain growth to form a thin PPy layer. At the same time, the pyrrole monomer dissolved in the solution also generated pyrrole radical cations, and then migrated to the surface of ATP and subsequently reacted with pyrrole radical cations to form granular PPy nanoparticles^46^.

The concentration of ATP clay was proved to have effects on the morphology, proportion of the components and the electrical conductivity of the nanocomposite. When the concentration of ATP clay was low (Fig. 2(c–e)), almost ATP were coated with PPy showing a beads-on-a-string morphology. When the concentration of ATP clay was high (Fig. 2(f–h)), the excess ATP could not be dispersed uniformly resulting in the aggregation of ATP, and many ATP clay were as same as original. In the XPS results, the N/Ag atomic ratio gradually decreased as the concentration of ATP increased. The variation on viscosity of the system may be responsible for the result. The viscosity of the system became higher when the feeding concentration of ATP increased, which would impede the migration of pyrrole radical cations, leading to slower speed of chain growth and lower yield of PPy. The influence of ATP concentration on the electrical conductivity is caused by two contrary effects of increasing ATP concentration. The increase in ATP concentration enhanced the compactness of the sample, which would help to enhance the electrical conductivity. But meanwhile, the increasing ATP concentration would decrease the electrical conductivity since it is an insulated material. Based on the above reasons, the variation trend for electrical conductivity that increased firstly and then decreased appeared. The abrupt increment for S-5 may cause by the highest content of Ag nanoparticles in the composite was certified by XPS.

In the application instance, PPy/Ag/ATP composite nanoparticles were explored to be used as multifunctional filler to prepare biodegradable PBS composite material. The unique properties that based on synergy effect and special morphology of the nanoparticles can affect the performance of the composite material. The as prepared PPy/Ag/ATP composite nanoparticles are not irregular mixture of PPy, Ag and ATP nanoparticles; on the contrary, they have unique structure (as shown in Fig. 2), which can improve the compatibility between ATP clay and PBS matrix thanks to the PPy layer presented on the surface of ATP. The enhancement in storage modulus of PBS-based composite is attributed to the reinforcing effect of PPy/Ag/ATP composite nanoparticles. It also reveals that PPy/Ag/ATP composite nanoparticles are well dispersed and have strong interactions with PBS matrix. The increasing content of PPy/Ag/ATP composite nanoparticles has little influence on the shift of tan δ peak as compared with that of pure PBS, but obvious effect on the decrease of the height of tan δ peak. The reduction in the height of tan δ peak results from the fact that the presence of PPy/Ag/ATP composite nanoparticles can give rise to stiffness improvement of PBS. The DMA results are similar with other fiber reinforced PBS composites^47^,^48^. The fibrous composite nanoparticles are well dispersed in PBS matrix and form three-dimensional network structure, which is in favour of improving interconnection between individual PPy/Ag/ATP composite nanoparticle and consequently imparting modest surface resistivity (10^6^ ~ 10^9^ Ω/◻). The excellent antibacterial effect owes to metallic Ag generated during the UV-induced polymerization. The antibacterial mechanism of silver nanoparticles is a complex response and proposed by the release of silver ions. It is reported that silver ions can interact with the bacterial cell, giving rise to the bacterial death^49^.

In conclusion, PPy/Ag/ATP composite nanoparticles with interesting beads-on-a-string morphology were successfully prepared by a facile UV-induced dispersion polymerization in 10 min. FTIR and XPS analysis confirmed that PPy in the nanocomposites was doped by NO_3_^−^, and the doping level was about 0.29. When they were used as multifunctional filler to prepare biodegradable PBS composite material, expectant properties such as good surface resistivity, improved storage modulus and outstanding antibacterial activity were obtained. These new imparted properties can broaden the applications of PBS in the fields of antibacterial and antistatic materials. In addition, instead of ATP clay, other substrates also can be applied to prepare composite containing PPy and Ag nanoparticles by UV-induce polymerization technique.

## Methods

### Materials

Pyrrole (Nacalai Tesque, Inc., Kyoto, Japan) was freshly distilled under pressure before use. Silver nitrate (AgNO_3_) and ethanol were purchased from Nacalai Tesque, Inc. (Kyoto, Japan) and used as received. All the reagents were of analytical grade. Attapulgite (ATP, Attagel 40) nanofibrillar clay was provided by BASF Co. Ltd. (New Jessey, USA).

### Preparation of PPy/Ag/clay composite nanoparticles

PPy/Ag/clay composite nanoparticles were prepared by UV-induced dispersion polymerization at various feeding amount of ATP. The composition used for preparing the composite is listed in Table 1. Initially, ATP clay was dispersed ultrasonically in 50 mL of deionized water for 30 min to obtain a colloidal network. Then the clay colloid was kept under vigorous stirring at 0 °C for another 30 min. After that, 1.0 mL of freshly distilled pyrrole was added and the mixture was kept under vigorous ultrasonic for 30 min in ice-cold condition. Subsequently, 10 mL of AgNO_3_ aqueous solution (containing 2.45 g of AgNO_3_) was added and the suspension kept under stirring for 5 min. The suspension was transferred to a glass vessel and placed 20 cm below the UV lamp (365 nm, 1000 W). UV-radiation was conducted for 10 min. After UV-induced polymerization was completed, the samples were filtered and washed several times with deionized water and ethanol, and then dried for 24 h at 40 °C under vacuum.

### Preparation of PBS-based biocomposite specimen

The mixture of commercial PBS and PPy/Ag/clay composite nanoparticles (S-3) with a ratio of 85:15 and 70:30 (w/w) was dried at 80 °C for 12 h before melt blending, respectively. The melt blending was carried out in a small kneading machine (CH-1100, Create Plastic Co., Ltd., Japan) at 135 °C for 10 min with a screw speed of 40 rpm. The extruded sample was cut into pellets and dried for next molding, which was performed using a conventional hot-press molding machine (Imoto Machinery Co., Ltd., Japan). The molding temperature was set at 135 °C and the size of the specimen was 100 mm × 100 mm × 1 mm. The prepared biocomposites were coded as 15% PBS composite and 30% PBS composite based on the mass fraction of PPy/Ag/ATP composite nanoparticles. For comparison, pure PBS specimen was prepared in the same conditions.

### Characterization

The morphologies of the nanoparticles were characterized by scanning electron microscope (SEM, Hitachi S-4300, Japan) and transmission electron microscope (TEM, Hitachi S-8100, Japan). The molded PBS-based biocomposite specimen was immerged in liquid nitrogen and then fractured. The fracture surface was observed by SEM. The chemical structures of the nanoparticles were confirmed with Fourier transform infrared (FT-IR, IRT-7000, Jasco, Japan) spectroscopy. X-ray diffraction (XRD) patterns were taken with a PANalytical X’Pert Pro X-ray diffractometer in the range of 2θ = 5–90°. X-ray photoelectron spectra (XPS) were recorded using a PHI 5000 Series XPS instrument. PPy/Ag/clay composite nanoparticles were shaped into circular pellets with a diameter of 13 mm to measure the electrical conductivity using RTS-8 four-point probe meter (Guangzhou Four-point Probe Meter Electronic Technology Co., Ltd) at ambient temperature. The thermal stability of the nanoparticles was tested by thermogravimetric analysis (TGA, Shimadzu DTG-60, Japan) at a heating rate of 10 °C/min from 25 to 800 °C under a nitrogen atmosphere. The surface resistivity of the PBS-based biocomposite specimen was measured using a resistivity meter (MCP-HT 450, Mitsubishi, Japan). Dynamic mechanical analysis (DMA) was done on RSA-G2 (TA Instruments, USA) in the three-point bending mode clamp under air at a frequency of 1 Hz and a strain amplitude of 0.1%. The specimens were analyzed from −60 °C–80 °C at a heating rate of 2 °C/min.

### Antibacterial activity test

For a quantitative evaluation, the antibacterial activity of 15% PBS composite specimen was tested according to the Japanese industrial standard, JIS Z 2801: 2010^45^. Three PBS-based biocomposite specimens and six pure PBS specimens (for the blank reference) with a size of 50 mm × 50 mm × 1 mm were prearranged. *Escherichia coli* NBRC 3972 and *Staphylococcus aureus* NBRC 12732 with a concentration of 2.5×10^5^ ~ 10×10^5^ cells/mL were used as the test inoculum. 0.4 mL of the test inoculum was pipetted onto the surface of specimen and covered with a piece of film that measured 40 mm × 40 mm for 24 h at 35 ± 1 °C with a relative humidity ≥90%. Immediately after inoculation, 10 mL of neutralizer was added to completely wash the specimen and a series of 10-fold dilutions were made. Then the dilutions were separately applied to plate count agar and a second incubation was performed at 35 ± 1 °C for 40 to 48 h. The number of colonies in the Petri dishes containing 30 to 300 colonies was counted and antibacterial activity was calculated using the following equation:


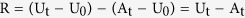


Where, R is the antibacterial activity; U_t_ and A_t_ is the average of the common logarithm of the number of viable bacteria recovered from the untreated and treated test specimens after 24 h, respectively. Generally, antibacterial activity is certified if R is >2.

## Additional Information

**How to cite this article**: Zang, L. *et al*. Preparation and application of conducting polymer/Ag/clay composite nanoparticles formed by *in situ* UV-induced dispersion polymerization. *Sci. Rep*. **6**, 20470; doi: 10.1038/srep20470 (2016).

## Figures and Tables

**Table 1:** The composition used for preparing the composite.

**Sample**	**ATP (g)**	**Pyrrole (mL)**	**ATP/pyrrole**^a^	**AgNO**_**3**_^**b**(g)^	**Size of Ag (nm)**	**Conductivity (S/cm)**
S-1	0.048	1.0	5:100	2.45	33	—
S-2	0.097	1.0	10:100	2.45	37	0.122
S-3	0.193	1.0	20:100	2.45	40	0.442
S-4	0.290	1.0	30:100	2.45	38	0.299
S-5	0.484	1.0	50:100	2.45	37	0.340

^a^mass ratio; ^b^ fixed 1:1 molar ratio of AgNO_3_ to pyrrole.

**Table 2:** XPS analysis of PPy/Ag/clay nanocomposites.

**Sample**	**C (atom%)**	**N (atom%)**	**O (atom%)**	**Ag (atom%)**	**Si (atom%)**	**N/Ag**
S-1	72.83	13.52	12.87	0.47	0.25	28.8
S-2	69.51	12.52	15.04	0.42	2.07	29.8
S-3	65.85	11.67	17.17	0.66	4.61	17.7
S-4	55.60	9.28	23.71	0.68	7.67	13.7
S-5	51.78	7.81	28.81	1.08	8.99	7.2

## References

[b1] Marambio-Jones C. & Hoek E. M. A review of the antibacterial effects of silver nanomaterials and potential implications for human health and the environment. J. Nanopart. Res. 12, 1531–1551 (2010).

[b2] Abreu A. S. . Antimicrobial nanostructured starch based films for packaging. Carbohydr. Polym. 129, 127–134 (2015). 26050897 10.1016/j.carbpol.2015.04.021

[b3] Lightcap I. V., Kosel T. H. & Kamat P. V. Anchoring semiconductor and metal nanoparticles on a two-dimensional catalyst mat. Storing and shuttling electrons with reduced graphene oxide. Nano Lett. 10, 577–583 (2010). 20055433 10.1021/nl9035109

[b4] Chun K. Y. . Highly conductive, printable and stretchable composite films of carbon nanotubes and silver. Nat. Nanotechnol. 5, 853–857 (2010). 21113161 10.1038/nnano.2010.232

[b5] Ijeri V. S. . An elegant and facile single-step UV-curing approach to surface nano-silvering of polymer composites. Soft Matter 6, 4666–4668 (2010).

[b6] Wallace G. G., Spinks G. M., Kane-Maguire L. A. P. & Teasdale P. R. Conductive Electroactive Polymers-Intelligent Polymer Systems. (CRC Press, London, 2009).

[b7] Attia M. F. . One-step UV-induced modification of cellulose fabrics by polypyrrole/silver nanocomposite films. J. Colloid Interface Sci. 393, 130–137 (2013). 23273672 10.1016/j.jcis.2012.11.008

[b8] Das D. . Nickel oxide/polypyrrole/silver nanocomposites with core/shell/shell structure: synthesis, characterization and their electrochemical behaviour with antimicrobial activities. Mater. Chem. Phys. 142, 61–69 (2013).

[b9] Jing S., Xing S., Yu L. & Zhao C. Synthesis and characterization of Ag/polypyrrole nanocomposites based on silver nanoparticles colloid. Mater. Lett. 61, 4528–4530 (2007).

[b10] Pinter E. . Characterization of polypyrrole-silver nanocomposites prepared in the presence of different dopants. J. Phys. Chem. B 109, 17474–17478 (2005). 16853234 10.1021/jp0517652

[b11] Yang X. & Lu Y. Preparation of polypyrrole-coated silver nanoparticles by one-step UV-induced polymerization. Mater. Lett. 59, 2484–2487 (2005).

[b12] Ayad M. M. & Zaki E. Synthesis and characterization of silver–polypyrrole film composite. Appl. Surf. Sci. 256, 787–791 (2009).

[b13] Babu K. F., Dhandapani P., Maruthamuthu S. & Kulandainathan M. A. One pot synthesis of polypyrrole silver nanocomposite on cotton fabrics for multifunctional property. Carbohydr. Polym. 90, 1557–1563 (2012). 22944416 10.1016/j.carbpol.2012.07.030

[b14] Breimer M. A., Sy S. & Sadik O. A. Incorporation of metal nanoparticles in photopolymerized organic conducting polymers: a mechanistic insight. Nano Lett. 1, 305–308 (2001).

[b15] Yagci Y., Jockusch S. & Turro N. J. Photoinitiated polymerization: advances, challenges, and opportunities. Macromolecules 43, 6245–6260 (2010).

[b16] Kobayashi N., Teshima K. & Hirohashi R. Conducting polymer image formation with photoinduced electron transfer reaction. J. Mater. Chem. 8, 497–506 (1998).

[b17] Singh A. . Photo-induced synthesis of polypyrrole-silver nanocomposite films on N-(3-trimethoxysilylpropyl) pyrrole-modified biaxially oriented polyethylene terephthalate flexible substrates. RSC Adv. 3, 5506–5523 (2013).

[b18] Singh A. . Electrochemical investigation of free-standing polypyrrole–silver nanocomposite films: a substrate free electrode material for supercapacitors. RSC Adv. 3, 24567–24575 (2013).

[b19] Ijeri V. S., Nair J. R., Gerbaldi C., Bongiovanni R. M. & Penazzi N. Metallopolymer capacitor in “one pot” by self-directed UV-assisted process. ACS Appl. Mater. Interfaces 2, 3192–3200 (2010). 20949927 10.1021/am1006639

[b20] Wu J. . High-performance polypyrrole nanoparticles counter electrode for dye-sensitized solar cells. J. Power Sources 181, 172–176 (2008).

[b21] Hakansson E. . Characterization of conducting polymer coated synthetic fabrics for heat generation. Synth. Met. 144, 21–28 (2004).

[b22] Huang B., Kang G. J. & Ni Y. Preparation of conductive paper by *in-situ* polymerization of pyrrole in a pulp fibre system. Pulp & Paper Canada 107, 38–42 (2006).

[b23] Zang L., Qiu J., Yang C. & Sakai E. Preparation and characterization of bayberry-like polypyrrole composites using functionalized mesoporous silica as *in situ* dopant. Int. J. Polym. Mater. Polym. Biomat. 64, 489–495 (2015).

[b24] Strandwitz N. C., Nonoguchi Y., Boettcher S. W. & Stucky G. D. *In situ* photopolymerization of pyrrole in mesoporous TiO_2_. Langmuir 26, 5319–5322 (2010). 20329722 10.1021/la100913e

[b25] Lim S. P., Pandikumar A., Lim Y. S., Huang N. M. & Lim H. N. *In-situ* electrochemically deposited polypyrrole nanoparticles incorporated reduced graphene oxide as an efficient counter electrode for platinum-free dye-sensitized solar cells. Sci. Rep. 4, 5305 (2014). 24930387 10.1038/srep05305 PMC4058879

[b26] Yang C. & Liu P. Polypyrrole/conductive mica composites: preparation, characterization, and application in supercapacitor. Synth. Met. 160, 768–773 (2010).

[b27] Jing S., Xing S. & Zhao C. Direct synthesis of PbS/polypyrrole core-shell nanocomposites based on octahedral PbS nanocrystals colloid. Mater. Lett. 62, 41–43 (2008).

[b28] Kobayashi Y. . Synthesis of metallic copper nanoparticles coated with polypyrrole. Colloid Polym. Sci. 287, 877–880 (2009).

[b29] Maeda S. & Armes S. P. Preparation and characterisation of novel polypyrrole–silica colloidal nanocomposites. J. Mater. Chem. 4, 935–942 (1994).

[b30] Lascelles S. F., McCarthy G. P., Butterworth M. D. & Armes S. P. Effect of synthesis parameters on the particle size, composition and colloid stability of polypyrrole-silica nanocomposite particles. Colloid Polym. Sci. 276, 893–902 (1998).

[b31] Damm C. & Münstedt H. Properties of silver-filled acrylate photopolymer layers prepared by a heterogeneous photocatalytic polymerisation reaction. Surf. Coat. Technol. 202, 5122–5126 (2008).

[b32] Campbell F. W. & Compton R. G. The use of nanoparticles in electroanalysis: an updated review. Anal. Bioanal. Chem. 396, 241–259 (2010). 19730834 10.1007/s00216-009-3063-7

[b33] Wakshlak R. B. K., Pedahzur R. & Avnir D. Antibacterial activity of silver-killed bacteria: the “zombies” effect. Sci. Rep. 5, 9555 (2015). 25906433 10.1038/srep09555 PMC5386105

[b34] Mikhalovsky S. V. . A comparative study of air-dry and water swollen flax and cotton fibres. RSC Adv. 2, 2868–2874 (2012).

[b35] Sakamoto M., Fujistuka M. & Majima T. Light as a construction tool of metal nanoparticles: synthesis and mechanism. J. Photochem. Photobiol. C 10, 33–56 (2009).

[b36] Efimov O. N. Polypyrrole: a conducting polymer; its synthesis, properties and applications. Russ. Chem. Rev. 66, 443 (1997).

[b37] Oh E. J., Jang K. S. & MacDiarmid A. G. High molecular weight soluble polypyrrole. Synth. Met. 125, 267–272 (2001).

[b38] Ghosh S., Bowmaker G. A., Cooney R. P. & Seakins J. M. Infrared and Raman spectroscopic studies of the electrochemical oxidative degradation of polypyrrole. Synth. Met. 95, 63–67 (1998).

[b39] Koji N. Infrared absorption spectroscopy. (Holden Day Inc, San Francisco, 1964).

[b40] Parikh A. N. . n-Alkylsiloxanes: from single monolayers to layered crystals. The formation of crystalline polymers from the hydrolysis of n-octadecyltrichlorosilane. J. Am. Chem. Soc. 119, 3135–3143 (1997).

[b41] Beamson G. & Briggs D. High resolution XPS of organic polymers: the scienta ESCA300 database. (John Wiley & Sons, Chichester, 1992).

[b42] Frost R. L. & Ding Z. Controlled rate thermal analysis and differential scanning calorimetry of sepiolites and palygorskites. Thermochim. Acta 397, 119–128 (2003).

[b43] Saha S. . Production of silver nanoparticles by a phytopathogenic fungus bipolaris nodulosa and its antimicrobial activity. Dig. J. Nanomater. Biostruct. 5, 887–895 (2010).

[b44] Tran H. V. . Synthesis, characterization, antibacterial and antiproliferative activities of monodisperse chitosan-based silver nanoparticles. Colloid Surf. A 360, 32–40 (2010).

[b45] Kourai H. . Antibacterial products-test for antibacterial activity and efficacy: JIS Z 2801: 2010. (Japanese Standards Association, Tokyo, 2010).

[b46] Yang C. . Nano-cladding of natural microcrystalline cellulose with conducting polymer: preparation, characterization, and application in energy storage. RSC Adv. 4, 40345–40351 (2014).

[b47] Muthuraj R., Misra M. & Mohanty A. K. Injection molded sustainable biocomposites from poly(butylene succinate) bioplastic and perennial grass. ACS Sustain. Chem. Eng. 3, 2767–2776 (2015).

[b48] Bin T. . Non-isothermal crystallization kinetics and dynamic mechanical thermal properties of poly(butylene succinate) composites reinforced with cotton stalk bast fibers. Thermochim. Acta 525, 141–149 (2011).

[b49] Gupta A., Maynes M. & Silver S. Effects of halides on plasmid-mediated silver resistance in Escherichia coli. Appl. Environ. Microbiol. 64, 5042–5045 (1998). 9835606 10.1128/aem.64.12.5042-5045.1998 PMC90966

